# 

**Published:** 1897-03

**Authors:** 


### CONTENTS.

ORIGINAL COMMUNICATIONS. PAGE
The Degenerate Jaws and Teeth. By EUGENE S. TALBOT, M.D., D.D.S. . . . . 141
The Work of James Edmund Garretson, A.M., M.D., D.D.S. By H. H. BURCHARD, M.D., D.D S. . . . . 151
A High Voltage Current will stop a Threatened Alveolar Abscess. By WILLIAM ROLLINS, Boston . . . . 155
Electrical Irritation of Fillings. By DR. A. H. PORTER, Philadelphia . . . . . 156
Annual Address. By DAVID W. CHEEVER, M.D., Boston . . . . 157

ABSTRACTS AND TRANSLATIONS.
The Decay of the Teeth . . . . 160

REPORTS OF SOCIETY MEETINGS.
American Dental Association . . . . 169
Academy of Stomatology . . . . 175
Central Dental Association of Northern New Jersey . . . . 184

EDITORIAL.
The Evolution and Abuse of the Serration . . . . 188

BIBLIOGRAPHY . . . . 192

OBITUARY.
Resolutions of Respect, Dr. George C. Brown . . . . 193
Dr. Francis Peabody . . . . 194
In Memoriam.—Dr. James A. Swasey . . . . 195

DOMESTIC CORRESPONDENCE.
Correction . . . . 197

NOTES AND COMMENTS . . . . 197

CURRENT NOTES.
Dental Society of the State of New York . . . . 200
Twelfth International Medical Congress . . . . 200
Odontographic Society of Chicago . . . . 202
The St. Louis Dental Society—Officers for 1897 . . . . 202
The Central Dental Association of Northern New Jersey . . . . 202

SELECTIONS.
Oxidation . . . . 203
Formalin Gelatin : A New Mode of Antiseptic Treatment . . . . 203

